# Economic modeling for improved prediction of saving estimates in healthcare costs from consumption of healthy foods: the Mediterranean-style diet case study

**DOI:** 10.29219/fnr.v63.3418

**Published:** 2019-09-17

**Authors:** Jason P.H. Jones, Mohammad M.H. Abdullah, Dallas Wood, Peter J.H. Jones

**Affiliations:** 1 RTI International, Durham, NC; 2 Department of Food Science and Nutrition, Kuwait University, Kuwait City, Kuwait; 3 Nutritional Fundamentals for Health, Vaudreuil-Dorion, QC, Canada

**Keywords:** nutrition economics, Mediterranean-style diet, economic modeling, Monte Carlo simulation, healthcare cost savings

## Abstract

**Background:**

By design, existing scenario-based nutrition economics studies on the financial benefits of healthy dietary behaviors generally report uncertainty in inputs and wide ranges of outcome estimates.

**Objectives:**

This modeling exercise aimed to establish precision in prediction of the potential healthcare cost savings that would follow a reduction in the incidence of cardiovascular disease (CVD) consistent with an increase in adherence to a Mediterranean-style diet (MedDiet).

**Design:**

Using a *Monte Carlo* simulation model on a cost-of-illness analysis assessing MedDiet adherence, CVD incidence reduction, and healthcare cost savings in the United States and Canada, short- and long-term cost savings that are likely to accrue to the American and Canadian healthcare systems were estimated using 20 and 80% increases in MedDiet adherence scenarios.

**Results:**

Increasing percentage of population adhering to a MedDiet by 20% beyond the current adherence level produced annual savings in CVD-related costs of US$8.2 billion (95% confidence interval [CI], $7.5–$8.8 billion) in the United States and Can$0.32 billion (95% CI, $0.29–$0.34 billion) in Canada. An 80% increase in adherence resulted in savings equal to US$31 billion (95% CI, $28.6–$33.3 billion) and Can$1.2 billion (95% CI, $1.11–$1.30 billion) in each respective country.

**Conclusion:**

Computational techniques with stochastic parameter inputs, such as the *Monte Carlo* simulation, could be an effective way of incorporating variability of modeling parameters in nutrition economics studies for improved precision in estimating the monetary value of healthy eating habits.

## Popular scientific summary

- Dramatic rates of major public health concerns, such as heart disease and cancer place great pressure on national economies worldwide.
- The Mediterranean diet is a classic example of healthy dietary habits and has consistently shown favorable impacts on the heart health.
- This economic model suggests a significant reduction in healthcare costs associated with the management of heart disease by following the dietary principles of the Mediterranean diet.

The skyrocketing health and economic burden of diet-related noncommunicable disorders, including obesity, type 2 diabetes, cardiovascular disease (CVD), and cancers, are highly recognized worldwide. For instance, management of CVD, the most prevalent and costly cause of mortality worldwide, accounting for over 30% of all deaths, was estimated to cost US$863 billion globally in 2010 and is projected to exceed a US$1 trillion by 2030 (1). Similarly, the economic burden of CVD is large in the United States, with an annual cost of over $300 billion based on 2013–2014 estimates (2) and in Canada, with an estimated cost of over Can$12 billion every year based on 2008 monetary figures (3). In Europe, the healthcare cost of CVD has been estimated at €210 billion per year (4).

Dietary behaviors consistent with the principles of healthy eating have the potential to improve the health and well-being of both individuals and societies in general, along with producing meaningful economic benefits (5). Nutrition economics studies have, especially over the past decade, successfully offered an important understanding of monetizing the value of healthcare responses to numerous healthy dietary habits (6–14). In this regard, using a scenario-based analysis, we have previously estimated annual healthcare and related cost savings that would range from US$1.0 to $62.8 billion in the United States and Can$41.9 million to $2.5 billion in Canada from the reduction in CVD incidence following the adoption of the Mediterranean-style diet (MedDiet) (10), a widely recommended dietary approach for prevention of chronic disease (15, 16). The MedDiet is classically high in fresh fruits, vegetables, whole grains, legumes, fish, and olive oil; moderate in dairy products; moderate to low in wine (mainly with meals); and low in red meat (17, 18).

It is generally accepted that most existing scenario-based nutrition economics studies report an inherent uncertainty in inputs and, consequently, wide ranges of resulting estimates, with no prediction as to where in the spectrum an actual outcome would be most likely to occur. Such inputs have traditionally been varied using sensitivity analyses, such as pessimistic through optimistic assumptions for key parameters. Applying a sensitivity analysis using the extremes of observed values does not fully assimilate the distributional information available for these parameters in the literature. The objective of the present modeling exercise was thus to establish precision in prediction of the potential short- and long-term savings in costs of healthcare and productivity loss that would arise from reduced incidence of CVD consequent to increased adherence to MedDiet as a classical example of healthy eating patterns.

## Methods

### Overview of the financial model design

This is an adapted version of a three-step variation of a cost-of-illness analysis, which we have previously presented (10), designed to 1) assess adherence to a MedDiet among adults in the United States and Canada, 2) estimate reduction in the incidence of CVD following increasing MedDiet adherence, and 3) impute annual healthcare cost savings following the reduction in CVD incidence. Previously, to account for the uncertainty factor in adherence to the MedDiet and reduction in the CVD rates across populations, a sensitivity analysis of four scenarios, including very pessimistic, pessimistic, optimistic, and ideal assumptions, has been implemented within each step (10). The present work extends on our original analysis in that it employs adherence scenarios relative to a baseline to capture increases in adherence to the MedDiet and a *Monte Carlo* simulation of subsequent CVD risk reduction parameters across studies in the literature. Updated CVD-related cost data were utilized for each country, where 10-year horizon time frame outcomes based on current trends allowed for long-term projections.

*Monte Carlo* simulation, or probability simulation, is a technique used to assess the effects of the distribution of input parameters on the distribution of the final result (19), by using repeated random draws from predetermined distributions to incorporate the statistical information as to the likelihood a parameter takes on different values. The key feature of a *Monte Carlo* simulation is that, unlike a classical sensitivity analysis, it allows for an outcome’s maximum likelihood or mean value to be recovered (20).

### Step 1: Adherence to the Mediterranean-style diet (% of population)

In the first step of this analysis, a distribution reflecting the percentages of both the American and Canadian adult populations who are likely to adhere to a MedDiet was obtained by extrapolating MedDiet score (MedScore) data from three US studies (21–23) (Table 1). MedScores are questionnaire-based scoring systems designed to determine the degree of adherence to the MedDiet (24). A simple average across the three US-based studies of interest (21–23) was used to construct a current ‘baseline’ MedDiet population adherence level in North American target populations. The majority of MedScore studies report percentages of populations that fell within three adherence tertiles, that is, low, medium, and high, each of which is composed of a range of MedScores reflecting degrees of MedDiet intakes. Other point-based MedScore systems exist; however, for consistency, only those using 5- and 9-point MedScores were used in this analysis.

**Table 1:** Summary on adherence to the Mediterranean-style diet in North American studies

Reference	Study design, place	Participants (*n*)	Dietary assessment tool	MedDiet adherence tertiles (% of participants)		
				Lowest	Medium	Highest
Ahmad et al. (21)	Prospective cohort (Women’s Health Study), United States	25,994	9-point MedScore	39	36.2	24.8
Park et al. (22)	Cross-sectional data (NHANES III, 1988–1994), United States	4,700	5-point MedScore	36.8	31.9	31.3
Tsivgoulis et al. (23)	Prospective cohort (REGARDS Study), United States	20,197	9-point MedScore	32.8	41.4	25.8

To determine the benefit of higher adherence to a MedDiet, two scenarios reflecting increases in current percentages of populations who follow the MedDiet by 20 and 80% were constructed, extending from a recent analysis by Scrafford et al. (25). Fractions of the population assumed to transition from low to medium, medium to high, and low to high were calculated and applied to a reduction in CVD incidence specific to their dietary changes. The current average MedScore level is 4.2 points of a 9-point MedScores (21–23), with 36 and 27% of the population reporting adherence equivalent to medium and high scores, respectively. Under the 20% increase scenario, the percentages of population required to shift from low to medium adherence and from medium to high adherence were 9.3 and 18.8%, respectively. Under the 80% scenario, the population percentages of low to medium and medium to high MedDiet adherences were 35.5 and 48.3%, respectively. It is important to note that the percentages of population shifting from low to high are included in each of these shifts; however, the CVD reduction impact is additive, such that the impact is not double counted. This is of importance when considering the 80% shift case, as it results in near-perfect conformance. Scrafford et al. noted the unrealistic nature of this scenario; however, stated that its usefulness lies in showing an upper bound of savings (25).

### Step 2: Reduction in the incidence of CVD

To associate the MedDiet adherence scenarios (step 1) with the reduction in CVD incidence, step 2 of this model employed data from five cohorts studies (21, 26–29), reporting adjusted hazard ratios (HRs) or relative risks (RRs) with corresponding 95% confidence intervals (CIs) (Table 2). These studies estimated the CVD reduction that followed upward shifts in the MedDiet consumption levels, from lowest to medium and from medium to highest intakes.

**Table 2:** Summary on reduction in cardiovascular disease rate from adherence to the Mediterranean-style diet in North American studies

References	Study design, place	Diet	Participants (*n*)	CVD reduction (lowest to medium, RR or HR)	CVD reduction, (lowest to highest, RR or HR)
Ahmad et al. (21)	Prospective cohort (Women’s Health Study), United States	MedDiet	25,994	0.77 (95% CI, 0.67–0.90)	0.72 (95% CI, 0.61–0.86)
Shikany et al. (26)	Prospective cohort (REGARDS Study), United States	MedDiet	3,562	0.91 (95% CI, 0.76–1.10)	0.78 (95% CI, 0.62–0.98)
George et al. (27)	Prospective cohort (WHI OS), United States	MedDiet	63,805	0.82 (95% CI, 0.70–0.97)	0.79 (95% CI, 0.67–0.94)
Gardener et al. (28)	Cohort (NOMAS), United States	MedDiet	1,559	0.72 (95% CI, 0.54–0.96)	0.75 (95% CI, 0.56–0.99)
Fung et al. (29)	Cohort (Nurses’ Health), United States	MedDiet	1,399	0.87 (95% CI, 0.77–0.99)	0.71 (95% CI, 0.62–0.82)

**Fig. 1:**
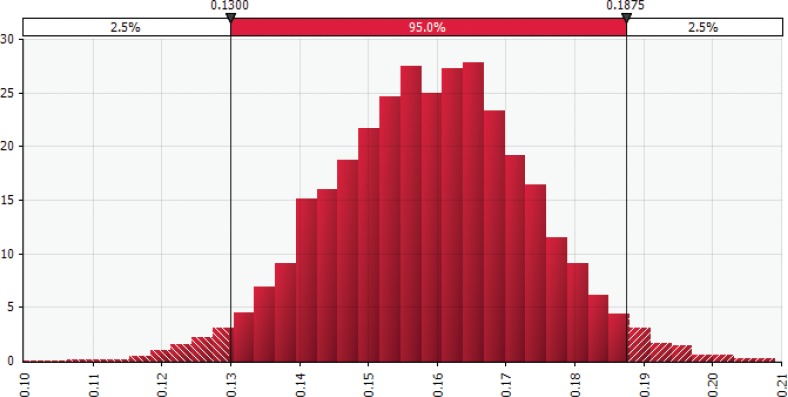
Generated joint probability density function for cardiovascular disease reduction rate from low to high Mediterranean-style diet adherence. Illustrates the distribution of 10,000 *Monte Carlo* simulation results for the joint probability density function for the cardiovascular disease (CVD) reduction rate. This distribution was constructed using the studies listed in Table 2 for low to high Mediterranean-style diet (MedDiet) adherence. Specifically, we computed the probability of obtaining a value from a particular study based on its population size as a proportion of the total participants. For each study, a normal distribution was assumed based on reported hazard ratio (HR) or relative risk (RR) and a calculated standard error based on the reported 95% confidence interval. The discrete distribution across studies and the normal distributions within each study represent the CVD reduction rate. Results generated by using the @RISK software for Microsoft Excel.

A joint probability distribution was constructed for the CVD reduction rate from adherence to the MedDiet, assuming a distribution across studies and within a given study. Using the studies listed in Table 2, probability of obtaining a value from a particular study was computed based on its population size as a proportion of the total participants. For each study, a normal distribution was assumed as a function of the reported HR or RR and a calculated standard error (SE) based on the reported 95% CIs. The discrete distribution across studies and normal distributions within each study represented the CVD reduction rate. These parameters were subsequently simulated 10,000 times using the @RISK software (Palisade, NY, USA) and produced the joint probability distribution illustrated in Fig. 1. This distribution shows a highly concentrated mean of 25% with a large right tail, with 90% of the observations falling between 22 and 28%.

### Step 3: Calculation of savings on healthcare costs

#### Short-termcost saving analysis

Step 3 of this analysis assessed both the short-term (or immediate) and long-term potential cost savings within the American and Canadian healthcare systems, separately, following the CVD incidence reduction. Healthcare costs typically comprise direct and indirect components, where the former refers to costs of the operation of hospitals, medication, and healthcare professionals, and the latter includes costs related to the productivity losses caused by mortality, illness, and disability. Applying a methodology that we outlined in detail previously (10), corresponding the reduction in disease cost to each 1% decrease in incidence of CVD, healthcare savings were calculated for each of the direct and indirect cost components following inflation of recent estimates associated with CVD in the United States (Table 3) and Canada (Table 4) to reflect their year 2017 dollar levels by using the US Department of Labor’s Health Care conusmer price index (CPI) (30) and Statistics Canada’s Health Care CPI (31), respectively.

**Table 3:** Summary of the healthcare costs attributed to cardiovascular disease in the United States (US$ billion)

Year		2013–2014^a^	2017^b^
**Direct costs**	Hospital inpatient stays	90.3	101
	Hospital emergency room visit	10.6	11.9
	Hospital outpatient or office-based provider visits	46.3	51.8
	Home healthcare	19.6	21.9
	Prescribed medicines	32.3	36.1
	Total direct	199.1	222.6
**Indirect costs**	Lost productivity/mortality	130.5	145.9
**Total CVD cost**		329.6	368.5

a Adapted from Benjamin et al. (2)*.*

b Current dollars based on monetary adjustments according to the medical care category of the US Department of Labor’s Health Care Consumer Price Index (30).

**Table 4:** Summary of the healthcare costs attributed to cardiovascular disease in Canada (Can$ million)

Year		2010^a^	2017^b^
**Direct costs**	Hospitals	6,128	6,434
	Drugs	4,957	5,205
	Physicians	1,910	2,005
	Formal caregiving	5.1	5.4
	Total direct	13,000	13,650
	Mortality	130.9	137.4
**Indirect costs**	Morbidity and long-term caring	513.3	539
	Total indirect	644.2	676.4
	**Total CVD cost**	13,644	14,326

a From the Economic Burden of Illness in Canada 2010 report (32).

b Current dollars based on adjustments according to Statistics Canada’s Health Care Consumer Price Index (31).

Data on costs of CVD in the United States were extracted from Benjamin et al. (2), who published a report on behalf of the American Heart Association incorporating the Medical Expenditure Panel Survey data with current demographic figures to produce the direct (hospital, home healthcare, and prescribed medicines) and indirect (lost productivity/mortality) cost estimates. Costs of circulatory system diseases in the United States were increased by 12.9% (or ~1.9%) per year between 2013 and 2017. Accordingly, the total CVD costs of US$329.6 billion in 2013–*2014* were assumed to grow to $368.5 billion in 2017 (Table 3). For Canada, using direct and indirect costs of disease data from the Economic Burden of Illness in Canada 2010 report (32), costs of the circulatory system diseases were increased by 6.3% (or ~0.8%) per year between 2010 and 2017. As such, the total CVD costs of Can$13.6 billion in 2010 were assumed to grow to $14.3 billion in 2017 (Table 4).

#### Long-term cost saving analysis

To estimate the discounted value of the MedDiet over the next years, a similar approach to the short-term analysis described above was followed. There are two differences in the methodology between the short-term and long-term analyses. First, direct and indirect CVD-related costs were forecasted for the next 10 years. Ideally, this would be done by using statistical techniques to infer the long-term growth patterns in CVD costs based on an extended time-series of CVD cost data. However, annual CVD cost data for the United States and Canada are not available for each year. Therefore, to achieve a forecast, we assumed that the CVD costs would grow over the subsequent 10 years at the rate of the healthcare cost inflation observed over the previous 10 years. The US Health Care CPI (30) indicates that the healthcare costs in the United States increased by 35.4%, or an average of 3.54% per year, between 2007 and 2017. This implies that the total CVD costs in the United States will increase from US$368.5 to $521.8 billion by 2027. Similarly, Statistics Canada’s Health Care CPI (31) indicates that healthcare costs in Canada increased by 13.4%, or an average of 1.3% per year, between 2007 and 2017, implying that the total CVD costs in Canada will increase from Can$14.4 billion in 2017 to $16.4 billion in 2027.

The second difference in the methodology for the long-term analysis is that we assumed that in the long run all costs are variable. As a result, a 1% decrease in CVD incidence would lead to a 1% reduction in all cost caregories, for example, reduction in hospital inpatient stays. Based on these assumptions, cost savings were estimated for each year from 2017 to 2027 for both the United States and Canada. The present values of these cummulative cost savings were then calculated assuming a 1.8% discount rate.

## Results

### Short-term cost savings

The short-term healthcare cost savings under the 20 and 80% increases in the percentages of population adherence scenarios in the United States and Canada are presented in Tables 5 and 6, respectively. For the United States, the mean annual cost savings reached US$8.2 billion (95% CI, $7.5–$8.8 billion) under the 20% scenario and US$31 billion (95% CI, $28.6–$33.3 billion) under the 80% scenario. For Canada,  the annual cost savings reached Can$0.32 billion  (95% CI, $0.29–$0.34 billion) under the 20% scenario and Can$1.2 billion (95% CI, $1.11–$1.30 billion) under the 80% scenario. In both countries under study, the 20 and 80% increases in percentages of population adherence to the current mean MedScore resulted in 2.2 and 8.4% decreases in CVD-related healthcare costs, respectively.

**Table 5:** Potential cost savings attributed to the reduction in cardiovascular disease rate with 20 and 80% upward shifts in current adherence to the Mediterranean-style diet in the United States (2017 US$ billion)^a^

	20% increase in adherence scenario	80% increase in adherence scenario
**Direct savings**		
Hospital inpatient stays	0.6 (0.5–0.6)	2.2 (2.0–2.3)
Hospital emergency room visits	0.1 (0.1–0.1)	0.3 (0.2–0.3)
Hospital outpatient or office-based provider visits	0.3 (0.3–0.3)	1.1 (1.0–1.2)
Home healthcare	0.8 (0.7–0.8)	2.9 (2.7–3.2)
Prescribed medicines	1.3 (1.2–1.4)	4.9 (4.5–5.2)
Total direct	3.0 (2.8–3.2)	11.3 (10.5–12.2)
**Indirect savings**		
Lost productivity/mortality	5.2 (4.8–5.6)	19.6 (18.1–21.1)
**Total savings**	8.2 (7.5–8.8)	31.0 (28.6–33.3)

a Data are means and 95% CIs of *Monte Carlo* simulation results for cost savings attributed to the reduction in CVD rate with adherence to the MedDiet in the United States under 20 and 80% increases in current adherence scenarios. Savings are reported by type of cost in billions of 2017 US dollars.

**Table 6:** Potential cost savings attributed to the reduction in cardiovascular disease rate with 20 and 80% upward shifts in current adherence to the Mediterranean-style diet in Canada (2017 Can$ billion)^a^

	20% increase in adherence scenario	80% increase in adherence scenario
**Direct savings**		
Hospitals	0.04 (0.03–0.04)	0.14 (0.13–0.15)
Drugs	0.19 (0.17–0.20)	0.70 (0.65–0.76)
Physicians	0.07 (0.07–0.08)	0.27 (0.25–0.29)
Formal caregiving	0.00 (0.00–0.00)	0.00 (0.00–0.00)
Total direct	0.29 (0.27–0.32)	1.11 (1.03–1.20)
**Indirect savings**		
Mortality	0.00 (0.00–0.01)	0.02 (0.02–0.02)
Morbidity and long-term caring	0.02 (0.02–0.02)	0.07 (0.07–0.08)
Total indirect	0.02 (0.02–0.03)	0.09 (0.08–0.10)
**Total savings**	0.32 (0.29–0.34)	1.20 (1.11–1.30)

a Data are means and 95% CIs of *Monte Carlo* simulation results for cost savings attributed to the reduction in CVD rate with adherence to the MedDiet in Canada under 20 and 80% increases in current adherence scenarios. Savings are reported by type of cost in billions of 2017 Canadian dollars.

**Fig. 2:**
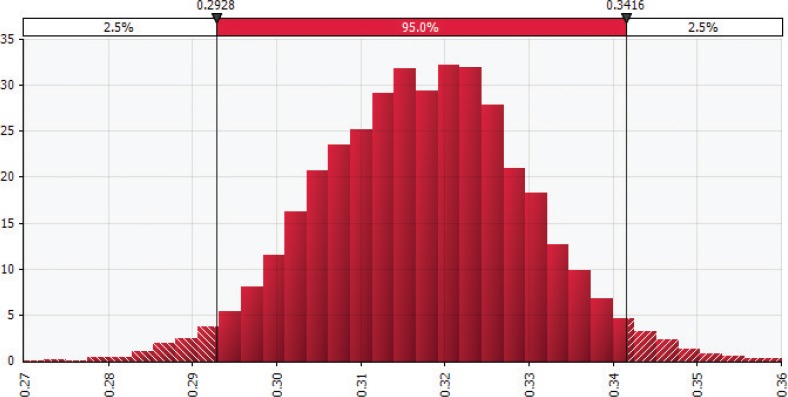
Distribution of *Monte Carlo* simulation results for the American healthcare cost savings given a 20% increase in adherence to Mediterranean-style diet, in year 2017 US$ billion. Illustrates the distribution of 10,000 *Monte Carlo* simulation results for estimated cost savings from adherence to Mediterranean-style diet (MedDiet) in the United States (measured in year 2017 US$ billion). These include direct cost savings, for example, from fewer prescribed medications and hospital stays, and indirect cost savings, for example, from lost productivity and mortality. We found 95% of simulation results fell between US$7.5 and $8.9 billion. Results generated by using the @RISK software for Microsoft Excel.

**Fig. 3:**
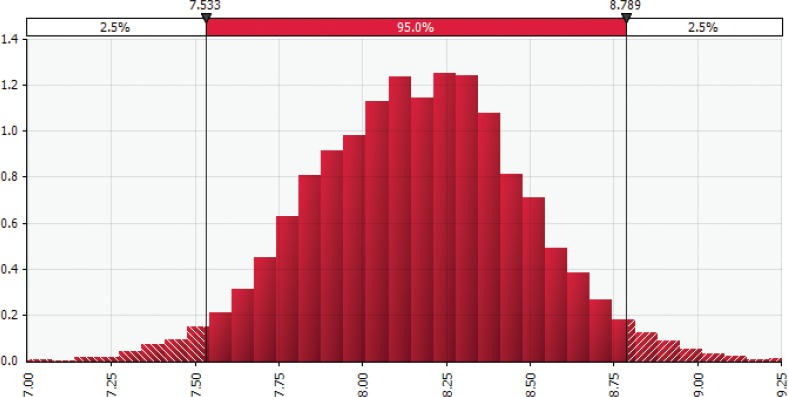
Distribution of *Monte Carlo* simulation results for the Canadian healthcare cost savings given a 20% increase in adherence to Mediterranean-style diet, in year 2017 Can$ billion. Illustrates the distribution of 10,000 *Monte Carlo* simulation results for estimated cost savings from adopting MedDiet in Canada (measured in year 2017 Can$ billion). These include direct cost savings, for example, from fewer prescribed medications and hospital stays, and indirect cost savings, for example, from lost productivity and mortality. We found 95% of simulation results fell between Can$0.29 and $0.34 billion. Results generated by using the @RISK software for Microsoft Excel.

The probability density functions, representing the total short-term cost savings under the 20% upward shift in adherence scenario for the United States and Canada, are shown in Figs. 2 and 3, respectively. The *X*-axis in each figure represents cost savings in billion dollars, while the *Y*-axis depicts the relative probabilities of the values within a range. As the parameter distributions for the MedDiet adherence and CVD reduction were assumed to be the same for both countries, the healthcare cost data shared the same distribution across countries. The model results found a significant central tendency due to the properties of the normal distribution incorporated into the *Monte Carlo* model through the CVD reduction parameter.

### Long-term cost savings

Results from the long-term analysis are provided for the United States in Table 7 and for Canada in Table 8. For the United States, we estimated that a cumulative 10-year mean cost savings under the 20 and 80% increases in population adherence scenarios would reach US$157.1 billion (95% CI, $144.9–$169.1 billion) and US$596.1 billion (95% CI, $550–$641.7 billion), respectively. Most of these savings are direct savings from hospitals. For Canada, a cumulative 10-year mean cost savings under the 20 and 80% adherence scenarios were estimated at Can$5.5 billion (95% CI, $5.1–$5.9 billion) and Can$20.9 billion (95% CI, $19.3–$22.5 billion), respectively. Most of these savings also come from direct savings, particularly in fixed hospital costs, and assume such costs increase over time.

**Table 7:** Potential discounted value of 10-year cost savings attributed to the reduction in cardiovascular disease rate with 20 and 80% upward shifts in current adherence to the Mediterranean-style diet in the United States (2017 US$ billion)^a^

	20% increase in adherence scenario	80% increase in adherence scenario
**Direct savings**		
Hospital inpatient stays	43.1 (39.7–46.3)	163.3 (150.7–175.8)
Hospital emergency room visits	5.1 (4.7–5.4)	19.2 (17.7–20.6)
Hospital outpatient or office-based provider visits	22.1 (20.4–23.7)	83.7 (77.3–90.1)
Home healthcare	9.3 (8.6–10.1)	35.4 (32.7–38.2)
Prescribed medicines	15.4 (14.2–16.6)	58.4 (53.9–62.9)
Total direct	94.9 (87.5–102.1)	360.1 (332.2–387.6)
**Indirect savings**		
Lost productivity/mortality	62.2 (57.4–66.9)	236 (217.8–254.1)
**Total savings**	157.1 (144.9–169.1)	596 (550.0–641.7)

a Data are means and 95% CIs of *Monte Carlo* simulation results for discounted value of 10-year cost savings attributed to the reduction in CVD rate with adherence to the MedDiet in the United States under 20 and 80% increases in current adherence scenarios. Savings are reported by type of cost in billions of 2017 US dollars.

**Table 8:** Potential discounted value of 10-year cost savings attributed to the reduction in incidence of cardiovascular disease with 20 and 80% upward shifts in current adherence to the Mediterranean-style diet in Canada (2017 Can$ billions)^a^

	20% increase in adherence scenario	80% increase in adherence scenario
**Direct savings**		
Hospitals	2.5 (2.3–2.7)	9.4 (8.6–10.1)
Drugs	2.0 (1.8–2.1)	7.6 (7.0–8.2)
Physicians	0.8 (0.7–0.8)	2.9 (2.7–3.1)
Formal caregiving	0.0 (0.0–0.0)	0.0 (0.0–0.0)
Total direct	5.2 (4.8–5.6)	19.9 (18.3–21.4)
**Indirect savings**		
Mortality	0.1 (0.0–0.1)	0.8 (0.7–0.8)
Morbidity and long-term caring	0.2 (0.2–0.2)	1.0 (0.9–1.1)
Total indirect	0.3 (0.2–0.3)	1.8 (1.6–1.9)
**Total savings**	5.5 (5.1–5.9)	20.9 (19.3–22.5)

a Data are means and 95% CIs of *Monte Carlo* simulation results for discounted value of 10-year cost savings attributed to the reduction in CVD rate with adherence to the MedDiet in Canada under 20 and 80% increases in current adherence scenarios. Savings are reported by type of cost in billions of 2017 Canadian dollars.

## Discussion

By employing a *Monte Carlo* simulation, the present modeling exercise showed improvement in prediction of the CVD-related healthcare cost savings that would arise in the United States and Canada from increases in percentages of populations’ adherence to a MedDiet. The outcomes of such an analysis will depend strongly on the input studies used to refect the target population. We selected a subset of available studies based on a number of characteristics; however, future studies should continue to broaden these inputs as is permitted by the modelling framework. In a previous scenario-based sensitivity analysis (10), we demonstrated total potential annual cost savings that ranged between US$1 and $63 billion in the United States, and Can$42 million and $2.5 billion in Canada, for a ‘very pessimistic’ scenario, a worst-case estimate that assumed only 5% of the population following a MedDiet and showing 10% reduction in CVD, through an ‘ideal scenario’, a best-case estimate of monetary savings when 50% of the population makes the dietary change and shows 60% reduction in CVD. In the present *Monte Carlo* design, the estimated 20–80% increases in percentages of populations who would follow the MedDiet were found to yield short-term annual cost savings of US$8.2 billion (95% CI, $7.5–$8.8 billion) to $31 billion (95% CI, $28.6–$33.3 billion) in the United States and Can$0.32 billion (95% CI, $0.29–$0.34 billion) to $1.2 billion (95% CI, $1.11–$1.3 billion) in Canada. Evidently, *even* though the healthcare cost data were updated since our last evaluation of this type was conducted in 2015 (10), the current saving estimates did fall within the uncertainty range of our previous work, demonstrating an impoved precision in prediction of the potential healthcare cost savings.

As mentioned earlier, the 80% scenario was introduced by Scrafford et al. (25) to denote a maximum bound, as the vast majority of the population is assumed to score within the high adherence category. Current MedScore studies have a poor representation of the complete population adherence distribution, as well as how the population is distributed within a tertile. This could lead to potential bias as a mean perserving shift assumes the population is uniformly distributed within each category. The approach taken in this research is an improvement from previous dichotumas approaches, though oppoutinities for improvement still exist. A representation of the MedDiet adherence as a continuous distribution would be ideal. Such underlying MedScore data are not currently available in the literature. Their availability, in addition to detailed nutrition behavior data, would allow for investigations into the impacts across individual food groups, dietary ingredients, or behavior changes, translating more effectively into policy and providing the public with better actionable information.

Close to US$400 billion in the United States and over Can$14 billion in Canada are spent on CVD management alone per annum (Tables 3 and 4). Given the potential value of the disease-related dollar savings that are observed with closer adherence to the MedDiet in this work, evidently, public health policies and interventions designed to encourage positive lifestyle choices assume a great economic importance for societies and governments alike. In establishing better food environments and increasing consumer MedDiet adherence beyond the current levels, initiatives should include such stakeholders as policy-making authorities, educators, researchers, healthcare professionals, food industrialists, and marketers. Governments at all levels, for example, can promote clear dietary guidelines, subsidy and food tax policies, and school food programs. Educators and healthcare providers could play a crucial role in ‘shop smart’ and media messages. Researchers may conduct national screening programs and socioecological model-based interventions. And the food industry can develop affordable products that will appeal to consumers. Such actions are expected to sustain healthy dietary behaviors and, consequently, lead to better quality of life while generating economic benefits at both the individual and societal levels.

The present modeling exercise expanded on our previous work in this area (10) to project the economic implications of the MedDiet beyond the short-term (or immdeiate) estimates and extending to a 10-year horizon. Including time trends across model inputs, such as dietary beliefs and behaviors, food industry structure, and healthcare costs, allows for increased model flexibility and more robust analyses, which are important for the public health sector and policy-making strategies. For example, the World Heath Organization reviewed country progress in creating enabling policy environments for promoting healthy diets and nutrition, where nutrition plans ranged from short-term action plans to 10-year nutrition strategies (33). Furthermore, variable uncertainty is expected to change over time due to changing demographics and income levels across regions, and technological innovation. A dynamic estimate of dietary adherence and disease incidence could also be incorporated, as the body of nutrition literature expands over time.

Uncertainty continues to be a challenging component of nutrition economics (34). The method proposed in this research hopes to provide a methodology to improve our ability to value nutrition interventions rather than relying on point estimates. *Monte Carlo* simulation methods have been widely used to capture uncertain inputs and model structure in other disciplines, such as hydrology (35), physics (36), ecology (37), and environmental sciences (38), resulting in a better understanding of how parameter sampling can lead to misleading results and the importance of testing a model under a range of input assumptions. Drawbacks to the *Monte Carlo* method are the significant amount of data required to construct input distributions and the reliability of models as being only as good as the inputs.

In conclusion, this modeling exercise underscores an importance of the *Monte Carlo* simulation as an effective way of incorporating variability of modeling parameters for improved precision in estimating the potential healthcare cost savings accosiated with healthy eating habits. Future studies of appropriate designs, including constructing economic models within, are likely to validate the findings of the present work. Such initiative, with the support of data from others, is expected to encourage public health authorities and stakeholder collaborations across jurisdictions in advocating greater adherence to the MedDiet as a key example of healthy dietary approaches among populations, leading to betterquality of life and economic value alike. Rather than only focusing on worst- and best-case scenarios, analyzing the statistical properties of nutrition intervention data and healthcare spending will improve policy-making prioritization of cost saving opportunities.

## Conflict of interest and funding

The authors have not received any funding or benefits from industry or elsewhere to conduct this study.

## References

[1] Bloom DE, Cafiero ET, Jané-Llopis E, Abrahams-Gessel S, Bloom LR, Fathima S, et al. The global economic burden of noncommunicable diseases. Geneva: World Economic Forum; 2011.

[2] Benjamin EJ, Virani SS, Callaway CW, Chamberlain AM, Chang AR, Cheng S, et al. Heart disease and stroke statistics-2018 update: a report from the American Heart Association. Circulation 2018; 137(12): e67–e492. doi: 10.1161/CIR.0000000000000573 29386200

[3] Public Health Agency of Canada Economic burden of illness in Canada, 2005–2008. Ottawa, ON: Public Health Agency of Canada; 2014.

[4] Wilkins E, Wilson L, Wickramasinghe K, Bhatnagar P, Leal J, Luengo-Fernandez R, et al. European Cardiovascular Disease Statistics 2017 Brussels: European Heart Network 2017 Available from: http://www.ehnheart.org/images/CVD-statistics-report-August-2017.pdf [cited 4 May 2018].

[5] Gyles CL, Lenoir-Wijnkoop I, Carlberg JG, Senanayake V, Gutierrez-Ibarluzea I, Poley MJ, et al. Health economics and nutrition: a review of published evidence. Nutr Rev 2012; 70(12): 693–708. doi: 10.1111/j.1753-4887.2012.00514.x 23206283

[6] Dall TM, Fulgoni VL 3rd, Zhang Y, Reimers KJ, Packard PT, Astwood JD Potential health benefits and medical cost savings from calorie, sodium, and saturated fat reductions in the American diet. Am J Health Promot 2009; 23(6): 412–22. doi: 10.4278/ajhp.080930-QUAN-226 19601481

[7] Bibbins-Domingo K, Chertow GM, Coxson PG, Moran A, Lightwood JM, Pletcher MJ, et al. Projected effect of dietary salt reductions on future cardiovascular disease. N Engl J Med 2010; 362(7): 590–9. doi: 10.1056/NEJMoa0907355 20089957 PMC3066566

[8] Gyles CL, Carlberg JG, Gustafson J, Davlut DA, Jones PJ Economic valuation of the potential health benefits from foods enriched with plant sterols in Canada. Food Nutr Res 2010; 54. doi: 10.3402/fnr.v54i0.5113. PMC2952539 20941328

[9] Schmier JK, Miller PE, Levine JA, Perez V, Maki KC, Rains TM, et al. Cost savings of reduced constipation rates attributed to increased dietary fiber intakes: a decision-analytic model. BMC Public Health 2014; 14: 374-2458-14-374. doi: 10.1186/1471-2458-14-374 PMC3998946 24739472

[10] Abdullah MM, Jones JP, Jones PJ Economic benefits of the Mediterranean-style diet consumption in Canada and the United States. Food Nutr Res 2015; 59: 27541. doi: 10.3402/fnr.v59.27541 26111965 PMC4481044

[11] Abdullah MM, Gyles CL, Marinangeli CP, Carlberg JG, Jones PJ Cost-of-illness analysis reveals potential healthcare savings with reductions in type 2 diabetes and cardiovascular disease following recommended intakes of dietary fiber in Canada. Front Pharmacol 2015; 6: 167. doi: 10.3389/fphar.2015.00167 26321953 PMC4531234

[12] Abdullah MM, Gyles CL, Marinangeli CP, Carlberg JG, Jones PJ Dietary fibre intakes and reduction in functional constipation rates among Canadian adults: a cost-of-illness analysis. Food Nutr Res 2015; 59: 28646. doi: 10.3402/fnr.v59.28646. 26652739 PMC4677277

[13] Abdullah MM, Jew S, Jones PJ Health benefits and evaluation of healthcare cost savings if oils rich in monounsaturated fatty acids were substituted for conventional dietary oils in the United States. Nutr Rev 2017; 75(3): 163–74. doi: 10.1093/nutrit/nuw062. PMC5914363 28158733

[14] Abdullah MMH, Marinangeli CPF, Jones PJH, Carlberg JG Canadian potential healthcare and societal cost savings from consumption of pulses: a cost-of-illness analysis. Nutrients 2017; 9(7). doi: 10.3390/nu9070793. PMC5537906 28737688

[15] Perk J, De Backer G, Gohlke H, Graham I, Reiner Z, Verschuren M, et al. European Guidelines on cardiovascular disease prevention in clinical practice (version 2012). The Fifth Joint Task Force of the European Society of Cardiology and Other Societies on Cardiovascular Disease Prevention in Clinical Practice (constituted by representatives of nine societies and by invited experts). Eur Heart J 2012; 33(13): 1635–701. doi: 10.1093/eurheartj/ehs092. 22555213

[16] Eckel RH, Jakicic JM, Ard JD, de Jesus JM, Houston Miller N, Hubbard VS, et al. 2013 AHA/ACC guideline on lifestyle management to reduce cardiovascular risk: a report of the American College of Cardiology/American Heart Association Task Force on Practice Guidelines. J Am Coll Cardiol 2014; 63(25 Pt B): 2960–84. doi: 10.1016/j.jacc.2013.11.003. 24239922

[17] Liyanage T, Ninomiya T, Wang A, Neal B, Jun M, Wong MG, et al. Effects of the Mediterranean diet on cardiovascular outcomes – a systematic review and meta-analysis. PLoS One 2016; 11(8): e0159252. doi: 10.1371/journal.pone.0159252. 27509006 PMC4980102

[18] Rosato V, Temple NJ, La Vecchia C, Castellan G, Tavani A, Guercio V Mediterranean diet and cardiovascular disease: a systematic review and meta-analysis of observational studies. Eur J Nutr 2019; 58(1): 173–91. doi: 10.1007/s00394-017-1582-0. 29177567

[19] Arenas DJ, Lett LA, Klusaritz H, Teitelman AM A Monte Carlo simulation approach for estimating the health and economic impact of interventions provided at a student-run clinic. PLoS One 2017; 12(12): e0189718. doi: 10.1371/journal.pone.0189718. 29284026 PMC5746244

[20] Hammersley JM, Handscomb DC Monte Carlo methods. London: Methuen; 1964.

[21] Ahmad S, Moorthy MV, Demler OV, Hu FB, Ridker PM, Chasman DI, et al. Assessment of risk factors and biomarkers associated with risk of cardiovascular disease among women consuming a Mediterranean diet. JAMA Netw Open 2018; 1(8): e185708. doi: 10.1001/jamanetworkopen.2018.5708. 30646282 PMC6324327

[22] Park YM, Zhang J, Steck SE, Fung TT, Hazlett LJ, Han K, et al. Obesity mediates the association between Mediterranean diet consumption and insulin resistance and inflammation in US adults. J Nutr 2017; 147(4): 563–71. doi: 10.3945/jn.116.243543. 28298537 PMC5368583

[23] Tsivgoulis G, Psaltopoulou T, Wadley VG, Alexandrov AV, Howard G, Unverzagt FW, et al. Adherence to a Mediterranean diet and prediction of incident stroke. Stroke 2015; 46(3): 780–5. doi: 10.1161/STROKEAHA.114.007894. 25628306 PMC4621211

[24] Zaragoza-Marti A, Cabanero-Martinez MJ, Hurtado-Sanchez JA, Laguna-Perez A, Ferrer-Cascales R Evaluation of Mediterranean diet adherence scores: a systematic review. BMJ Open 2018; 8(2): e019033-2017-019033. doi: 10.1136/bmjopen-2017-019033. PMC5855302 29478018

[25] Scrafford CG, Bi X, Multani JK, Murphy MM, Schmier JK, Barraj LM Health Economic evaluation modeling shows potential health care cost savings with increased conformance with healthy dietary patterns among adults in the United States. J Acad Nutr Diet 2019; 119(4): 599–616. doi: 10.1016/j.jand.2018.10.002. 30591404

[26] Shikany JM, Safford MM, Bryan J, Newby PK, Richman JS, Durant RW, et al. Dietary patterns and Mediterranean diet score and hazard of recurrent coronary heart disease events and all-cause mortality in the REGARDS study. J Am Heart Assoc 2018; 7(14). doi: 10.1161/JAHA.117.008078. PMC6064845 30005552

[27] George SM, Ballard-Barbash R, Manson JE, Reedy J, Shikany JM, Subar AF, et al. Comparing indices of diet quality with chronic disease mortality risk in postmenopausal women in the Women’s Health Initiative Observational Study: evidence to inform national dietary guidance. Am J Epidemiol 2014; 180(6): 616–25. doi: 10.1093/aje/kwu173. 25035143 PMC4157698

[28] Gardener H, Wright CB, Gu Y, Demmer RT, Boden-Albala B, Elkind MS, et al. Mediterranean-style diet and risk of ischemic stroke, myocardial infarction, and vascular death: the Northern Manhattan Study. Am J Clin Nutr 2011; 94(6): 1458–64. doi: 10.3945/ajcn.111.012799. 22071704 PMC3252546

[29] Fung TT, Rexrode KM, Mantzoros CS, Manson JE, Willett WC, Hu FB Mediterranean diet and incidence of and mortality from coronary heart disease and stroke in women. Circulation 2009; 119(8): 1093–100. doi: 10.1161/CIRCULATIONAHA.108.816736. 19221219 PMC2724471

[30] Bureau of Labor Statistics Measuring price change in the CPI: Medical care. United States Department of Labor Available from: https://www.bls.gov/cpi/factsheets/medical-care.htm [cited 4 May 2018].

[31] Statistics Canada Health Care Consumer Price Index http://www23.statcan.gc.ca/imdb/p2SV.pl?Function=getSurvey&SDDS=2301#a4.

[32] Public Health Agency of Canada Economic burden of illness in Canada, 2010. Ottawa, ON: Public Health Agency of Canada; 2018.

[33] Global nutrition policy review 2016–2017: country progress in creating enabling policy environments for promoting healthy diets and nutrition. Geneva: World Health Organization; 2018 Licence: CC BY-NC-SA 3.0 IGO.

[34] Lenoir-Wijnkoop I, Dapoigny M, Dubois D, van Ganse E, Gutierrez-Ibarluzea I, Hutton J, et al. Nutrition economics – characterising the economic and health impact of nutrition. Br J Nutr 2011; 105(1): 157–66. doi: 10.1017/S0007114510003041. 20797310 PMC3023144

[35] Kuczera G, Parent E Monte Carlo assessment of parameter uncertainty in conceptual catchment models: the Metropolis algorithm. J Hydro 1998; 211(1–4): 69–85.

[36] Lewis A, Bridle S Cosmological parameters from CMB and other data: a Monte Carlo approach. Phys Rev D 2002; 66(10): 103511. doi: 10.1016/S0022-1694(98)00198-X.

[37] Wintle BA, McCarthy MA, Volinsky CT, Kavanagh RP The use of Bayesian model averaging to better represent uncertainty in ecological models. Conserv Biol Ser 2003; 17(6): 1579–90. doi: 10.1111/j.1523-1739.2003.00614.x.

[38] Wang ZH, Bou-Zeid E, Au SK, Smith JA Analyzing the sensitivity of WRF’s single-layer urban canopy model to parameter uncertainty using advanced Monte Carlo simulation. J Appl Meteorol Climatol 2011; 50(9): 1795–814. doi: 10.1175/2011JAMC2685.1.

